# Immediate Pain Relief Elicited After Radiosurgery for Classical and Symptomatic Trigeminal Neuralgia

**DOI:** 10.7759/cureus.4777

**Published:** 2019-05-30

**Authors:** Alessandra Gorgulho, Nzhde Agazaryan, Michael Selch, Bruno Fernandes de O Santos, Antonio De Salles

**Affiliations:** 1 Neurosurgery, Hospital do Coração, Sao Paulo, BRA; 2 Radiation Oncology, University of California, Los Angeles, USA; 3 Neurosurgery, Federal University of Sergipe, Aracaju, BRA; 4 Neurosurgery, University of California, Los Angeles, USA

**Keywords:** immediate pain relief, trigeminal neuralgia, radiosurgery

## Abstract

Background

Immediate relief following radiosurgery for trigeminal neuralgia (TN) has been observed in a minority of cases.

Objective

Our goals were to determine the occurrence of immediate pain relief as real vs. placebo effect and to search for factors associated with this desirable outcome.

Methods

Between January 2003 and June 2008, 150 patients were treated with radiosurgery for classical or symptomatic TN. A commercially available linear accelerator (Novalis®, BrainLab) device was used to deliver 90 Gy to the root-entry zone with a 4- or 5-mm collimator. Pain outcomes were graded using a four-point scale. Complications were recorded through standardized follow-up evaluations. Treatment plans were retrieved and brainstem/trigeminal nerves were retrospectively re-contoured using standard anatomical landmarks. Dose-volume histograms were used to calculate the volume of brainstem/trigeminal nerve receiving 20%, 30%, and 50% of the prescribed radiation doses.

Results

Twenty-five (19.84%) patients presented with immediate pain relief, defined as pain cessation within 48 hours post-radiosurgery. Kaplan-Meier analysis showed that good/excellent pain outcomes were sustained and significantly better in the immediate pain relief group (*p *= 0.006) compared to non-immediate relief. Univariate and multivariate logistic regression analyses failed to show the correlation between brainstem/trigeminal nerve volumes, trigeminal nerve-pontine angle, prior surgical procedures, TN etiology, age, gender, and immediate pain relief. Neither post-radiosurgery complications nor recurrence rates were different between groups.

Conclusion

Immediate pain relief leads to sustained relief and patients present significantly better pain outcomes in comparison to those without immediate relief. The mechanism triggering immediate relief is still unknown and did not correlate with the volume of brainstem/trigeminal nerve receiving pre-specified doses of radiation.

## Introduction

Stereotactic radiosurgery (SRS) became an attractive modality within the armamentarium of treatment for trigeminal neuralgia due to its minimally invasive nature. The major drawback of radiosurgery is the time delay to trigger pain relief, precluding its indication for patients suffering severe acute pain attacks. The latency time to achieve pain relief varies from immediate to up to six months post-radiosurgery, averaging about six weeks [1-9].

Several authors suggest that pain relief rates are higher as the radiosurgery doses to the root entry zone and to the brainstem are increased [10-12]. Facial numbness, the most common post-radiosurgery complication, is also more frequently reported when delivering higher doses of radiation to the root entry zone (REZ) [11,13-18]. This evidence does not prove if the increased likelihood to obtain better pain control with higher doses to the REZ is clinically significant. Moreover, some authors reported that the relation between nerve volume and dose (ratio of dose to nerve volume and integral dose) may play a role in TN recurrence [19-20]. There is no randomized clinical trial comparing effectiveness and side effects among the different radiosurgery protocols and, as a result, the location of the treatment isocenter remains controversial. It is uncertain whether radiosurgery protocols could be modified to provide faster pain relief and higher rates of desirable pain relief.

Immediate pain relief is not a standard defined term in the radiosurgery literature. It describes pain cessation within a few days following the procedure, rather than immediate pain relief after radiation delivery is concluded. Since immediate pain relief is seldom observed, this outcome following SRS has not been the focus of many previous studies [21-22]. Two important questions arise considering this topic: 1) Is immediate pain relief real or a placebo effect following radiation delivery? 2) Are there specific factors associated with this outcome?

In an attempt to generate a feasible hypothesis that would justify properly designed clinical trials for evaluation of immediate relief, we retrospectively reviewed the data of the TN cohort submitted to radiosurgery. Our main hypothesis in regards to prognostic factors was that increased volumes of brainstem receiving 20%, 30%, or 50% of total radiation dose would lead to a higher incidence of immediate pain relief. We also hypothesized that a more acute angle between the trigeminal nerve and the pons would imply on a larger volume of brainstem submitted to high radiation doses, therefore correlating with immediate pain relief.

## Materials and methods

Study design and patient population

This study is a cohort of trigeminal neuralgia patients treated with radiosurgery between January 2003 and June 2008. This period was selected because the collection of information about immediate pain relief began in 2003. From 150 patients treated within the analyzed period, there were missing data on 24 (16%) patients. Analysis was performed on the remaining 126 patients. Seven of them were retreated with SRS, and the data of the second treatment was not included in the analysis. This study was approved by our Institutional Review Board. Participant’s consent for this study was not required since all subjects have completed all research-related interventions. Immediate pain relief was defined as complete pain cessation within 48 hours after the radiosurgery procedure.

Radiosurgery planning and delivery

Since 2003, the CISS sequence (2-mm slice thickness, at the region of interest) has been added to the TN planning protocol. Standard imaging for SRS planning includes the T1 sequence with contrast (1 mm slice thickness), the T1 without contrast sequence (3-mm slice thickness through whole brain) and the T1 coronal without contrast (1mm slice thickness). MRI images are acquired in a 1.5 T Sonata MRI (Siemens AG, Erlangen, Germany) and fused to the 1.5 mm slice thickness CT scan (Marconi Medical Systems, Mission Viejo, CA).

**Figure 1:**
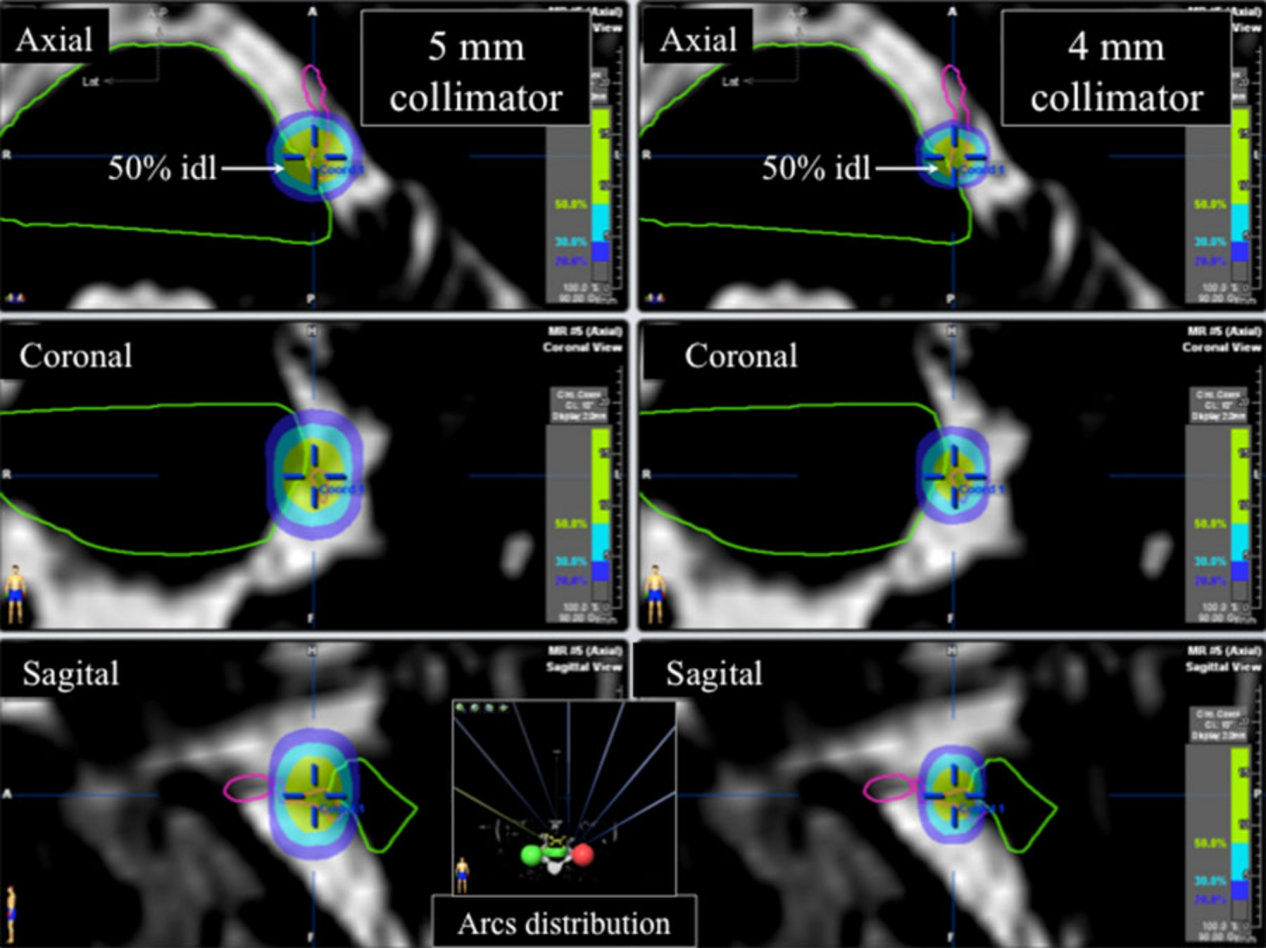
Radiosurgery plan Dose distribution of radiosurgery plans shown in axial, coronal and sagittal plans on CISS MRI sequence: a 5-mm collimator plan is shown in the left columns, while a 4-mm collimator is shown in the right columns. The arc distribution is presented in the lower central portion of the figure. The seven equally distributed arcs are displayed in coronal view.

The radiosurgery treatment has been described in detail in the prior publication [2]. The MRI scan was obtained the morning of the treatment day. The stereotactic frame was attached to the skull under local anesthesia and the CT scan was obtained. The CT was fused to the MRI for localization and three-dimensional treatment planning was carried out with a commercially available system (Brainscan 5.3 or iPlan 4.1, BrainLAB AG, Feldkirchen, Germany). Isocenter placement coincided with the REZ region of the trigeminal nerve. All patients were treated to a maximal dose of 90 Gy with the 50% isodose line tangential to the pontine surface. The 5-mm collimator was used in 118 (93.65%) cases and the 4-mm in eight (6.35%) cases (Figure 1). Since January 2008, all TN radiosurgeries have been done with the 4-mm collimator. After satisfactory planning was obtained, the patient was brought to the treatment room. Radiation was delivered by a dedicated 6MV linear accelerator (Novalis®, BrainLAB AG, Feldkirchen, Germany) and total treatment time lasted about 45 minutes. After radiation delivery was complete, the frame was detached from the head and the patient was released home.

Follow-up protocol

Patients remained on medications until pain relief was noticed and then medication was titrated to none, if possible. The information was prospectively collected by standardized follow-up questionnaires completed during clinic visits or by phone interviews. Patients come for the first follow-up at three months post-radiosurgery and at six and 12 months during the first year. Follow-up is updated once a year after that. Pain outcomes were measured in a four-grade points pain scale, described as follows:

1: No relief

2: Less than 50% pain relief

3: More than 50% pain relief or pain abolition with medication intake (good outcome)

4: Pain free and medication free (excellent outcome)

Grade 1 and 2 outcomes were considered failure.

Dose-volume histograms and trigeminal nerve-pontine angle measurements

**Figure 2:**
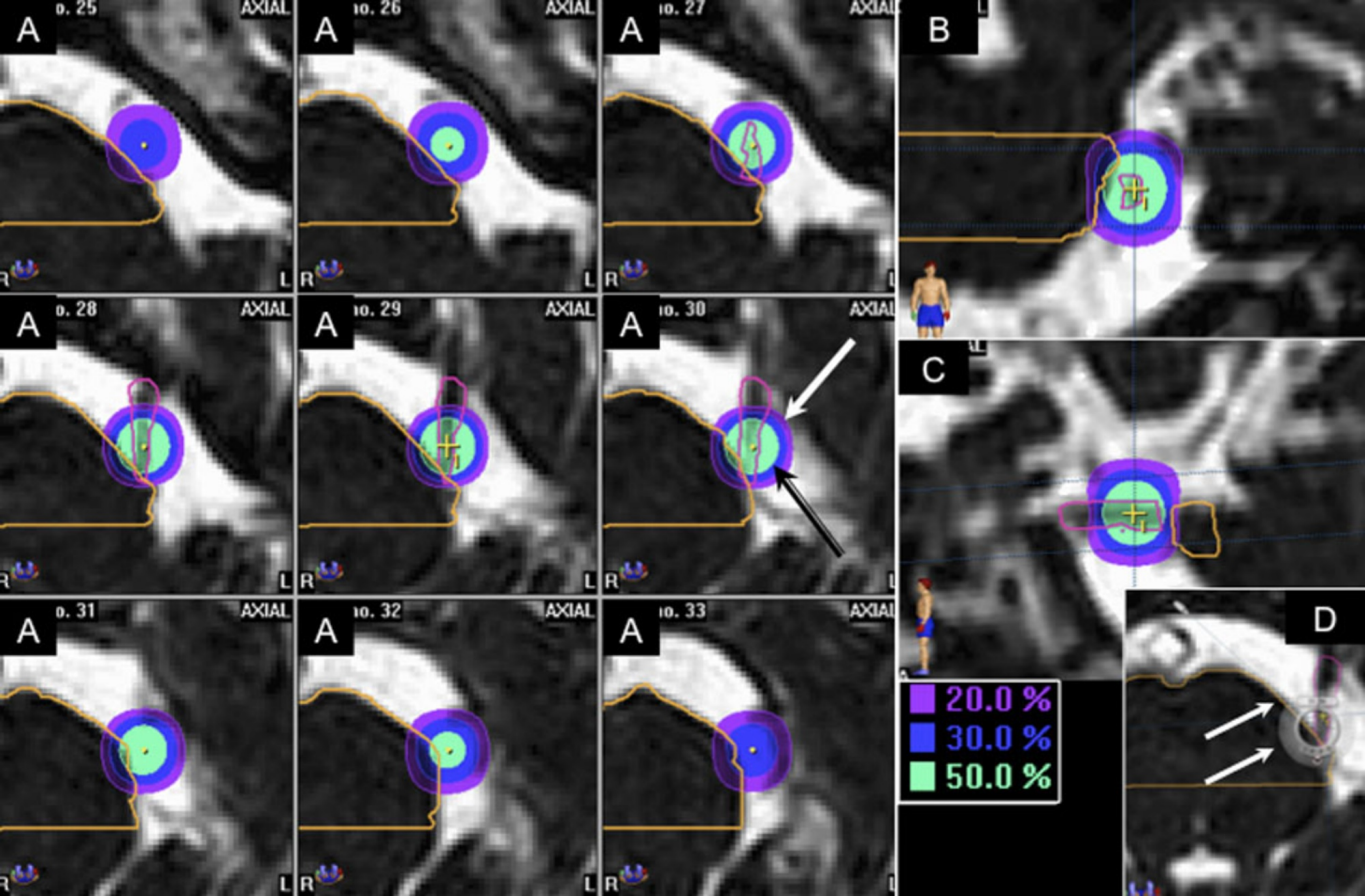
Dose distribution of the 20%, 30%, and 50% isodose lines (IDL) on the radiosurgery plan Definition of brainstem and trigeminal nerve volumes used for calculation of the dose volume histograms showing the dose distribution of the 20%, 30%, and 50% isodose lines (IDL) on the radiosurgery plan. A: axial CISS MRI sequence showing contours on nine slices. The black arrow points to the dose distribution of the 50% IDL while the white one points to the 20% IDL distribution. B and C: coronal and sagittal CISS MRI sequences of the brainstem and trigeminal nerve contours. D: The white arrows point to the digital compass positioned at the convergence of the nerve and the pons, where all measurements were taken.

The radiosurgery plans were retrieved; the brainstem and treated trigeminal nerve were re-contoured by a single author (AAG) using axial CISS sequences. The volume of irradiated tissue is minimal and to assure standardization of the contour of the structures being analyzed, the brainstem and the nerve were retrospectively re-contoured using the following anatomical landmarks as limits:

a) Brainstem: vertical extension was limited to a total of 11 slices (22 mm). The central slice was the axial slice where the isocenter was positioned and an additional five slices above and below this level. A straight horizontal line was traced at the cerebellopontine angle as the posterior brainstem limit for contouring, while the anterior pontine contours were set at pons anatomical limits. (Figure 2)

b) Trigeminal Nerve: only the cisternal portion starting at the emergence from the pons until the bone petrous margin was contoured, excluding the portion of the nerve inside the Meckel’s Cave.

The grid size for dose-volume histograms (DVH) calculation was set at 1mm. The volumes of the brainstem and trigeminal nerve receiving 20%, 30% and 50% of the total radiation dose were calculated from the DVH excel derived table.

The angle between the trigeminal nerve and the pontine surface was measured with the use of a digital compass positioned at the bottom of the angle in the axial slice coinciding with the isocenter positioning (Figure 2D).

Statistical analysis

The statistical software (SAS, version 9.1, SAS Institute, Cary, NC) was used in the analysis. Excellent and excellent/good pain outcome actuarial rates were compared between the immediate and non-immediate pain relief groups using the Kaplan-Meier method. Univariate and multivariate analyses of the biologically plausible prognostic factors that could lead to immediate relief were performed using logistic regression (Wald chi-square probability test). Variables analyzed were: patient age, etiology of trigeminal neuralgia, history of prior surgical procedures, trigeminal nerve-pontine angle, brainstem, and trigeminal nerve volumes irradiated by 20%, 30%, and 50% radiation dose.

To provide a complete report of the radiosurgery results in both groups, post-radiosurgery complications were also compared between the two groups using logistic regression. Variables included in the follow-up questionnaire were analyzed and comprised: facial numbness, facial paresthesias, decreased corneal reflex, irritated eyes, anesthesia dolorosa, facial palsy, hearing loss, and ataxia. Recurrence in both groups was analyzed using Cox's proportional hazards model.

## Results

There were 25 (19.84%) patients who met the criteria of immediate pain relief while the remaining 101 (80.16%) either achieved pain control later or failed radiosurgery. The mean patient age was 65.5 ± 13.9 years. There were 72 (57.1%) females and 54 (42.9%) males. A total of 110 (87.3%) patients had a diagnosis of classical trigeminal neuralgia. Radiosurgery was the first surgical procedure in 104 (81.7%) cases; 16 (12.7%) had radiosurgery as the second surgical procedure, while another 7(5.5%) cases have had two prior procedures. Detailed demographic data according to immediate pain relief status are described in Table 1. The mean time to achieve pain control in the group without immediate pain relief was 2.61 ± 2.47 months for those with good pain outcome and 3.18±3 months for those with excellent pain outcome.

**Figure 3:**
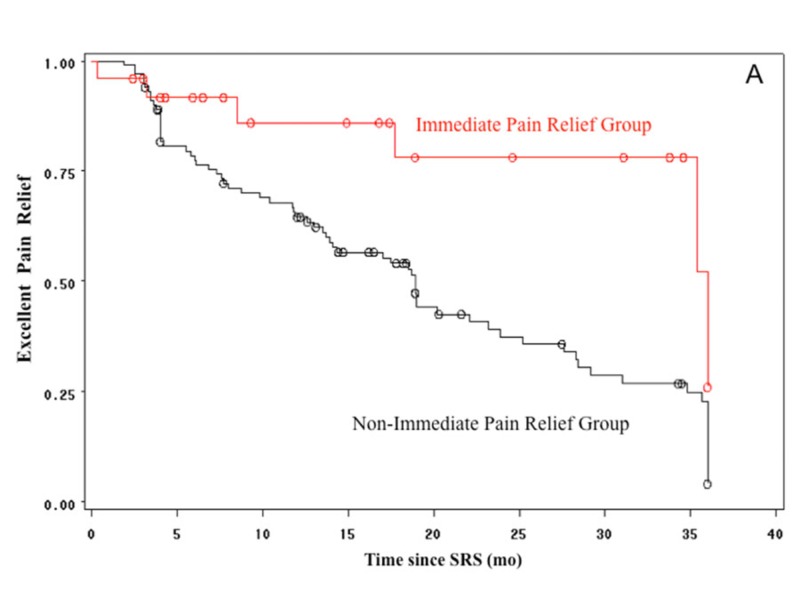
Kaplan-Meier curves showing excellent pain relief rate Kaplan-Meier curves showing in excellent pain relief rate in the immediate pain relief (red) and non-immediate pain relief (black) groups. From 126 patients, 68 (54%) presented with excellent pain control.

**Figure 4:**
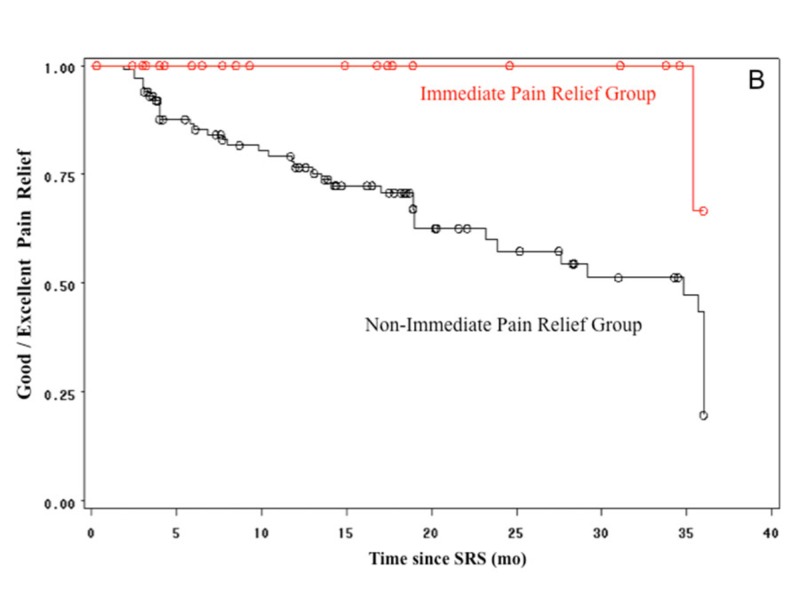
Kaplan-Meier curves showing good/excellent pain relief rate Kaplan-Meier curves showing good/excellent pain relief rate in the immediate pain relief (red) and non-immediate pain relief (black) groups. From 126 patients, 68 (54%) and 35 (28%) presented with excellent and good pain control, respectively.

Kaplan-Meier curves of the patients presenting good (*n *= 35, 28%) and/or excellent (*n* = 68, 54%) pain relief after radiosurgery were analyzed using follow-up at 36 months as the cut-off time point. The Kaplan-Meier curves of the group with immediate pain relief showed the sustainability of pain relief. The Kaplan-Meier analysis also demonstrates a significantly better pain outcome in the group with immediate pain relief vs. non-immediate relief using excellent relief or excellent/good relief as the outcome variables (log-rank test, *p *= 0.009 and 0.006, respectively; Figures 3-4). Since the number of patients with longer follow-up is reduced, the cut-off time at 36 months was chosen in order to show a more valid picture of pain outcomes in both groups.

Univariate analysis of potential factors associated with immediate pain relief occurrence is shown in Table 2. Although none of the variables shown significantly affected the incidence of immediate pain relief, history of prior surgical procedures approached significance. Previously, the surgical procedure was treated in the univariate analysis as both numerical and dichotomous variable with similar results. The different brainstem volumes were treated as continuous variables and the standardized coefficient of each radiation dose was used as the variable in the logistic equation.

Due to the high correlation among brainstem and trigeminal volumes, the multivariate analysis never had more than one brainstem and trigeminal volumes entered concomitantly with the other variables. Multivariate analysis using the backward selection model failed to disclose any statistically significant covariates. Univariate logistic regression results of post-radiosurgery complications using immediate pain relief as outcome variable are described in Table 3.

**Figure 5:**
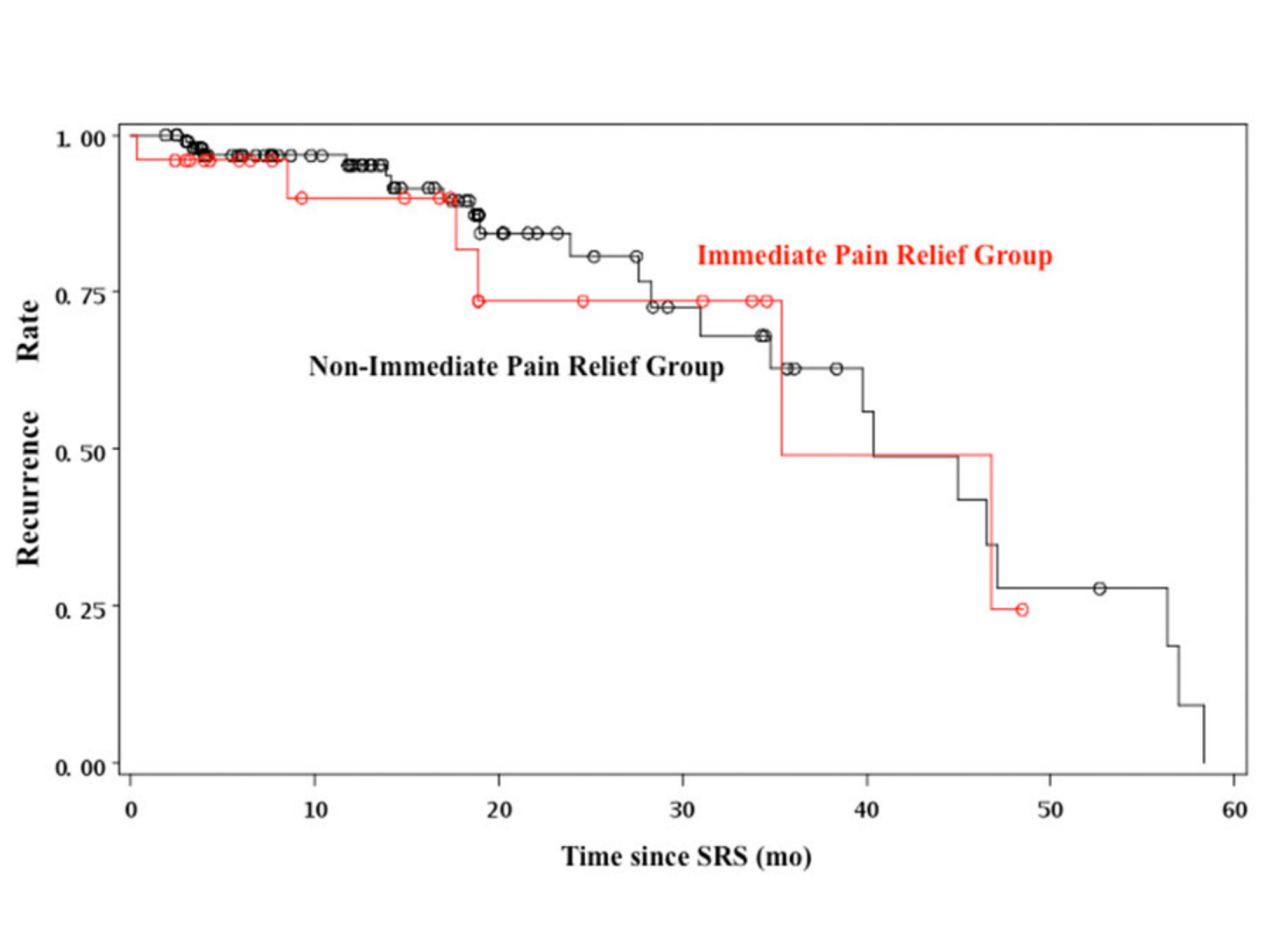
Cox proportional hazards plot of recurrence rates in the immediate pain relief (red) and non-immediate pain relief (black) groups Cox proportional hazards plot of recurrence rates in the immediate pain relief (red) and non-immediate pain relief (black) groups. As evidenced by the overlap of the curves, there was no significant difference in the recurrence rates on both groups.

Recurrence rates were calculated for both groups (Figure 5). Recurrence was defined as any drop from the best level of pain relief ever achieved after radiosurgery. This means that a patient presenting with excellent pain relief and later on requiring medication to maintain pain control was rated as recurrence. The recurrence hazard ratio for immediate vs. non-immediate relief was 0.906 (95% CI: 0.55-1.50). Pain control drop from excellent or good to poor occurred in only 10 (7.93%) of the 126 patients. Out of these 10 cases, four patients underwent repeated radiosurgery, one underwent microvascular decompression and the remaining five were managed solely with medication. No significant differences in the incidence of either complications or recurrences were observed.

## Discussion

The Kaplan-Meier analysis showed that immediate pain relief is not a placebo effect. Somewhat to our surprise, good and excellent pain outcomes were significantly more frequent among those who presented with immediate pain relief (Figure 3). Remarkably, immediate pain relief outcome did not lead to an increased incidence of facial numbness or other complications. Tuleasca et al. evaluated the impact of latency to pain relief in 497 patients with classical trigeminal neuralgia [23]. Pain response was categorized according to three time intervals from SRS: ≤48 hours, >48 hours to ≤30 days, >30days. Their results corroborate ours concerning immediate pain control, confirming this is not placebo response. They did not observe higher recurrence rates among the three groups analyzed.

Many series in the literature demonstrated a correlation between higher rates of overall pain relief and increased incidence of facial numbness [1,17-18,24-25]. Interestingly, immediate pain relief did not appear to require additional injury to the trigeminal pathway since complications rates were not different between immediate and non-immediate pain relief groups. A higher incidence of facial numbness among “late” responders (>30 days) was observed in the Tuleasca series, different from ours [23]. This observation actually reinforces our hypothesis that immediate pain relief occurrence would not require more extensive demyelination of the trigeminal pathway.

The absence of higher incidence of post-radiosurgery complications and the direction of the trend between immediate pain relief occurrence and volume of brainstem receiving 20%, 30%, or 50% of the total radiation dose does not corroborate our initial hypothesis. We hypothesized that an increased volume of brainstem receiving a given threshold dose of radiation would account for an increased incidence of immediate pain relief elicited by radiosurgery, mimicking some correlations described by other authors regarding better pain outcomes and dose to the root entry zone and brainstem [10-13,24-25].

The angle between the pons and brainstem offered the anatomical substrate to our hypothesis, justifying why similar protocols would result in ultimately different brainstem volumes submitted to high doses of radiation between both groups. Our analyses confirmed that trigeminal nerve volume has no significant effect. Nevertheless, good and/or excellent pain outcomes were significantly higher in the immediate relief group. This “dichotomization” of outcomes suggests that different mechanisms modifying pain sensory information within the trigeminal pathway may account for immediate versus sustained pain relief observed in these cases.

Experimental work delivering ionizing radiation to peripheral nerves showed that abolishment of nervous conduction is clearly dependent on the total radiation dose. It is also well known that peripheral nerves are highly resistant to ionizing radiation [26]. Conflicting results can be explained by the diversity of nerve preparations, radiation doses and rates, electrophysiology technique and time interval between radiation delivery and electrophysiology recording. Schwarz and Fox hypothesized that stimuli block would be the result of indirect destruction of ionic channels, mainly sodium [27]. The minimum radiation dose triggering the decrease of the sodium current ranged between 60 and 100 Gy.

One important aspect involved in the time course of response of trigeminal neuralgia pain to radiosurgery could be related to the amount of radiation reaching the actual target, i.e., the site in the trigeminal root entry zone where the demyelination is present. Our data showed that when we could confirm that the REZ was reached by the maximal radiation dose, as observed by contrast enhancement in the follow-up MRIs, patients presented with better pain outcomes [24]. Therefore, disruption of the possible short circuit was realized. Possibly the triggering of immediate relief may be dependent on the chance of enough demyelinated areas within the nerve to be located within the dose distribution of the higher radiation dose delivery.

As the current thickness of the targeting MRI and CT scans are between 1 and 1.5 mm, the best possible targeting accuracy is of 0.75 mm based solely on the imaging factor. We also need to consider that the expected accuracy of the stereotactic frame is 1.5 mm. This has been shown on a multicentric study for functional neurosurgery that included our own center data and has also been previously reported by others [28-29]. Therefore the possibility of suboptimal maximal dose delivery is real. This is more so if one also includes the radiation delivery device accuracy of approximately 0.5 mm. Having a trigeminal nerve of approximately 3 mm in diameter and a likely short circuit of less than 0.5 mm embedded in its REZ, it might be expected, at least in 80% of the cases, that the maximal dose does not reach the short circuit. Moreover, the radiation beam on time lasts about 45 minutes using our protocol and device.

The trigeminal nerve is also surrounded by cerebrospinal fluid within the pre-pontine cistern. There may be sub-millimeter oscillation of the nerve throughout the respiratory cycle. Although our target is fairly close to the pons, where a lesser extent of nerve oscillation should be expected, this faint mobility may impact in the overall final total radiation dose actually delivered to the nerve. Therefore, immediate pain relief is not observed in the majority of the patients, as they don’t have the disruption of this circuit realized at the time of the radiation. Our findings that both brainstem/REZ and trigeminal nerve tissue volumes receiving 20%, 30%, or 50% of the maximal radiation dose did not show association to immediate relief occurrence points out in favor to this proposed hypothesis. A random variation in the amount of radiation delivery, due to natural nerve movement and/or radiosurgery technical accuracy limitations, would explain why only in a minority of the cases a sufficiently high dose of radiation sufficient to trigger immediate relief is actually delivered to the demyelinated area of the nerve.

A reconciliatory theory about the pathogenesis of trigeminal neuralgia has been summarized [30]. The peripheral postulated mechanism is the observed ability of focal areas of demyelination to generate ectopic action potentials, besides the distortion of sensorial input to the central nervous system. Partial lesion of the peripheral portion of the nerve as achieved with radiation, radiofrequency, balloon compression, glycerol injection, and partial section leads to a substantial decrease in the overload of afferent sensory stimuli. Different from the other surgical techniques where an abrupt disruption of the sensory transmission is triggered by the procedure, the injury elicited by radiation implies in a process leading to sub-total damage and partial block of sensory information over time. Our lack of knowledge about how this partial injury to the trigeminal nerve elicited by radiation leads to pain control after an average delay of 4-6 weeks makes even more speculative a suggestion on how immediate relief could be elicited by radiation. Animal experiments established that the ability of radiation to completely block nervous conduction is dose-dependent. It was also shown that there is minimal required time latency to elicit conduction block independently of the dose rate and conditional upon the delivery of a minimal total radiation dose, which is in accordance with the 24-48 hours time delay observed in patients presenting with “immediate” pain control acutely after radiosurgery.

## Conclusions

Immediate pain relief leads to sustained relief and patients present significantly better pain outcomes in comparison to those without immediate relief. The mechanism triggering immediate relief is still unknown and did not correlate with the volume of brainstem/trigeminal nerve receiving pre-specified doses of radiation.
